# The possible role of hypoxia in the affected tissue of relapsed clubfoot

**DOI:** 10.1038/s41598-022-08519-z

**Published:** 2022-03-15

**Authors:** Tomas Novotny, Adam Eckhardt, Martina Doubkova, Jarmila Knitlova, David Vondrasek, Eliska Vanaskova, Martin Ostadal, Jiri Uhlik, Lucie Bacakova, Jana Musilkova

**Affiliations:** 1 Department of Orthopaedics, University J.E. Purkinje and Masaryk Hospital, Usti nad Labem, Czech Republic; 2 Department of Histology and Embryology, Second Faculty of Medicine, Charles University, Prague, Czech Republic; 3 Institute of Physiology of the Czech Academy of Sciences, Prague, Czech Republic; 4 Second Faculty of Medicine, Charles University, Prague, Czech Republic; 5 Faculty of Physical Education and Sport, Charles University, Prague, Czech Republic; 6 Department of Orthopaedics, University Hospital Bulovka, Charles University, Prague, Czech Republic

**Keywords:** Paediatric research, Musculoskeletal system, Pathogenesis, Physiology, Angiogenesis

## Abstract

Our aim was to study the expression of hypoxia-related proteins as a possible regulatory pathway in the contracted side tissue of relapsed clubfoot. We compared the expression of hypoxia-related proteins in the tissue of the contracted (medial) side of relapsed clubfoot, and in the tissue of the non-contracted (lateral) side of relapsed clubfoot. Tissue samples from ten patients were analyzed by immunohistochemistry and image analysis, Real-time PCR and Mass Spectrometry to evaluate the differences in protein composition and gene expression. We found a significant increase in the levels of smooth muscle actin, transforming growth factor-beta, hypoxia-inducible factor 1 alpha, lysyl oxidase, lysyl oxidase-like 2, tenascin C, matrix metalloproteinase-2, matrix metalloproteinase-9, fibronectin, collagen types III and VI, hemoglobin subunit alpha and hemoglobin subunit beta, and an overexpression of *ACTA2*, *FN1*, *TGFB1*, *HIF1A* and *MMP2* genes in the contracted medial side tissue of clubfoot. In the affected tissue, we have identified an increase in the level of hypoxia-related proteins, together with an overexpression of corresponding genes. Our results suggest that the hypoxia-associated pathway is potentially a factor contributing to the etiology of clubfoot relapses, as it stimulates both angioproliferation and fibroproliferation, which are considered to be key factors in the progression and development of relapses.

## Introduction

Clubfoot is one of the most common congenital deformities affecting the musculoskeletal system^1^. Its global prevalence is reported to be approximately 1 per 1000^2^; however, the rates vary among countries and geographical areas. Although clubfoot is a frequent orthopaedic disorder, and although some environmental, developmental and genetic factors that may contribute to its development have been described^3^, its etiology is still unclear. In addition, little is known at cellular and molecular level about specific pathways in the affected tissues of clubfoot, both before treatment and after relapses. A noticeable number of relapses occur even after initial treatment by the Ponseti method, which is now used worldwide, usually with great effectiveness^4^. These relapses are sometimes linked with more severe cases of clubfeet; however, roughly 1/3 of the relapses occur mainly due to poor compliance with the treatment^5^.

A study of possible pathologies of cells and their products that can lead to altered composition of the extracellular matrix (ECM) provides an important way to explore the pathologic tissue condition. However, only a few studies have provided such data on clubfoot and clubfoot related tissues^6^. To learn more about this condition, we made a study of changes in protein composition by comparing the contracted, i.e. medial (M-) side, tissue and the non-contracted, i.e. lateral (L-) side, tissue of relapsed clubfoot. In our previous study, performed on a different set of patients, we described the increase in fibrosis-associated proteins, such as type III, V and VI collagen, transforming growth factor-beta-induced protein (TGF-βIP), asporin and tenascin C content and expression^7^. Furthermore, we have detected a significant increase in the microvessel and arteriole density in the contracted M-side of the relapsed clubfoot tissue, which was connected to an increase in pro-angiogenic factors^8^. These pathways are identical with those described in other fibroproliferative and angioproliferative diseases, such as Dupuytren’s contracture and Peyronie’s disease^9,10^.

Diseases manifesting with fibroproliferation and angioproliferation are often associated with tissue hypoxia and related cell signaling^11–13^. The aim of the present study was to investigate the presence of hypoxia-related markers in the contracted tissue of relapsed clubfoot and to relate these findings with well-known cellular pathways in other diseases. The findings can provide important information about pathologic conditions in clubfoot tissue and can help to unravel the etiology of clubfoot relapses. Such knowledge may contribute to better future outcomes and even to the development of therapeutic strategies.

## Results

Tissue samples from medial (M-)side and lateral (L-)side of the foot were obtained from ten patients (nine boys, one girl; mean age of 53.4 months) with idiopathic clubfoot relapsed after unsuccessful Ponseti method treatment (Table 1). These tissues were evaluated for selected markers of tissue hypoxia and related proteins. Data obtained from the samples of contracted M-side tissue are compared with the non-contracted L-side tissue. The following hypoxia-related markers were evaluated: alpha smooth muscle actin (SmActin; *ACTA2* gene), transforming growth factor-beta (TGF-β; *TGFB1* gene), hypoxia-inducible factor 1 alpha (HIF1A; *HIF1A* gene), fibronectin (Fibronectin; *FN1* gene), lysyl oxidase (LOX), lysyl oxidase-like 2 (LOXL2), tenascin C (TN-C), matrix metalloproteinase-2 and -9 (MMP-2, MMP-9; *MMP2*, *MMP9* genes), hemoglobin subunit alpha (HBB) and beta (HBA1); with the addition of collagen type III (COL3), and type VI (COL6).

**Table 1:** Detailed data of 10 patients (sample donors) with a relapse of idiopathic congenital clubfoot.

Patient	Gender	Dimeglio clubfoot classification	Age during sample acquisition	Ponseti casting	Type of surgery	Previous surgeries	Clubfoot family anamnesis
1	M	III	38	10	PR	AT	N
2	M	III	44	10	PR	AT	N
3	M	III	60	5	MK	N	Y
4	M	III	34	6	PR	AT + R	N
5	M	III	75	5	PR	AT	N
6	F	III	52	5	MK	AT	N
7	M	IV	54	8	PR	AT	N
8	M	III	54	6	PR	AT	Y
9	M	IV	55	6	PR	AT	N
10	M	IV	68	6	PR	AT	N
Patients total: 10	1 × F 9 × M	7 × III 3 × IV	Mean 53.4 months (SD = 11.96)	Mean 12.3 (SD = 18.6)	2 × MK 8 × PR	1 × AT + R 8 × AT 1 × N	2 × Y 8 × N

The parents of these patients were generally non-compliant with the Ponseti regime, or were unable to maintain proper treatment by applying an abduction bar, or even failed to come to scheduled examinations. *M* male, *F* female, *AT* achilles tendon tenotomy, *AT + R* achilles tendon tenotomy + retenotomy, *MK* McKay, *PR* posteromedial release, *N* no, *Y* yes.

### Immunohistochemical quantification

**Figure 1:**
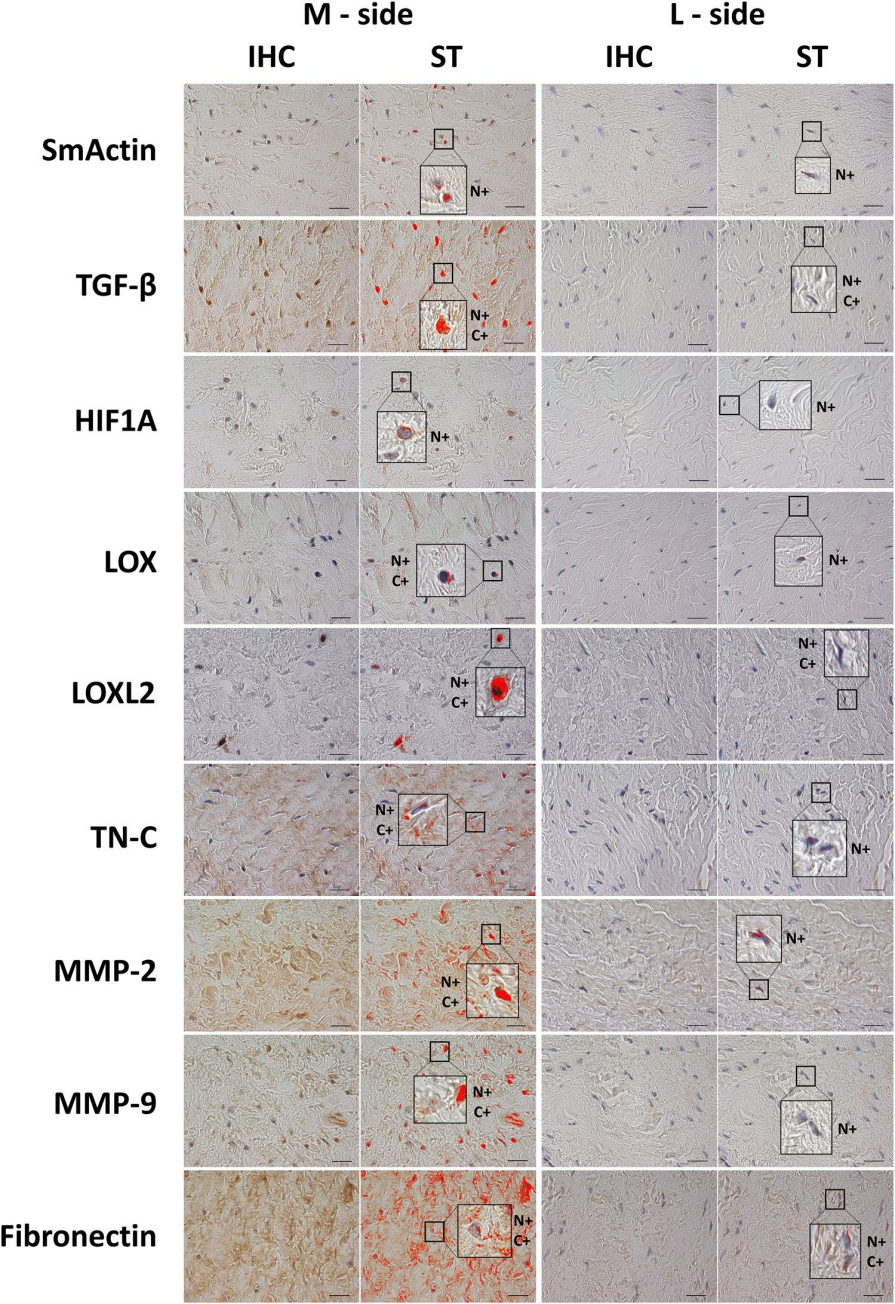
Demonstrative images of immunohistochemical staining and signal detection after the signal thresholding. Used antibodies: Anti-SmActin, Anti-TGF-β, Anti-HIF1A, Anti-LOX, Anti-LOXL2, Anti-TN-C, Anti-MMP-2, Anti-MMP-9, Anti-Fibronectin. The red areas in the ST columns represent the pixels analyzed after the thresholds were set. After applying the threshold settings, an image analyzer was used to measure the percentage of the area with a positive signal from the total area. *IHC* Immunohistochemistry, *ST* Signal Threshold, *N+* Nuclear positivity, *C+* Cytosolic positivity. Scale bar = 20 μm.

**Figure 2:**
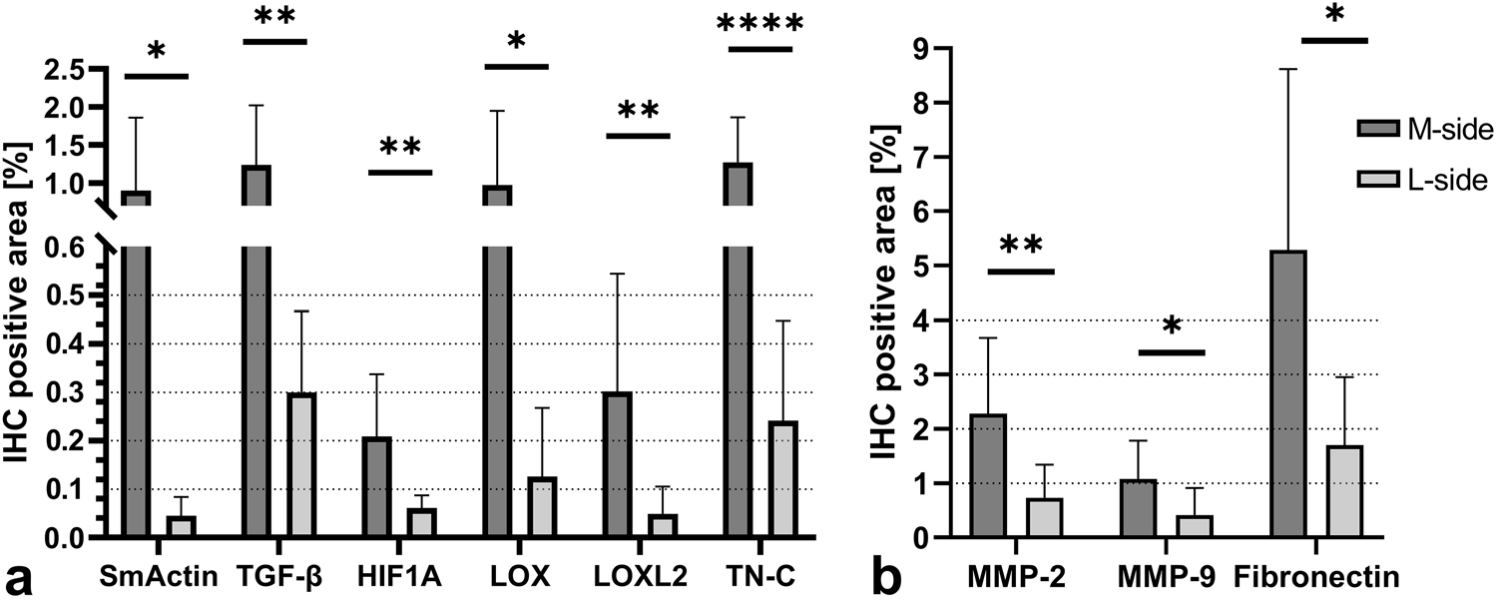
Immunohistochemical antibody detection. The percentage of (**a**) SmActin, TGF-β, HIF1A, LOX, LOXL2 and TN-C positive area and (**b**) the percentage of MMP-2, MMP-9 and Fibronectin positive area after immunohistochemical (IHC) detection**.** Data are presented as mean ± SD (n = 10). Values connected by lines differ significantly from each other (*p < 0.05, **p < 0.01, ***p < 0.005, ****p < 0.0005).

There was an increase in the percentage of the SmActin positive area (p = 0.0251), the TGF-β positive area (p = 0.0057), the HIF1A positive area (p = 0.0071), the LOX positive area (p = 0.0221), the LOXL2 positive area (p = 0.0207), the TN-C positive area (p = 0.0009), the MMP-2 positive area (p = 0.0097), the MMP-9 positive area (p = 0.0334) and the Fibronectin positive area (p = 0.0111) in the M-side compared to the L-side (Figs. 1, 2).

### Real-time PCR

**Figure 3:**
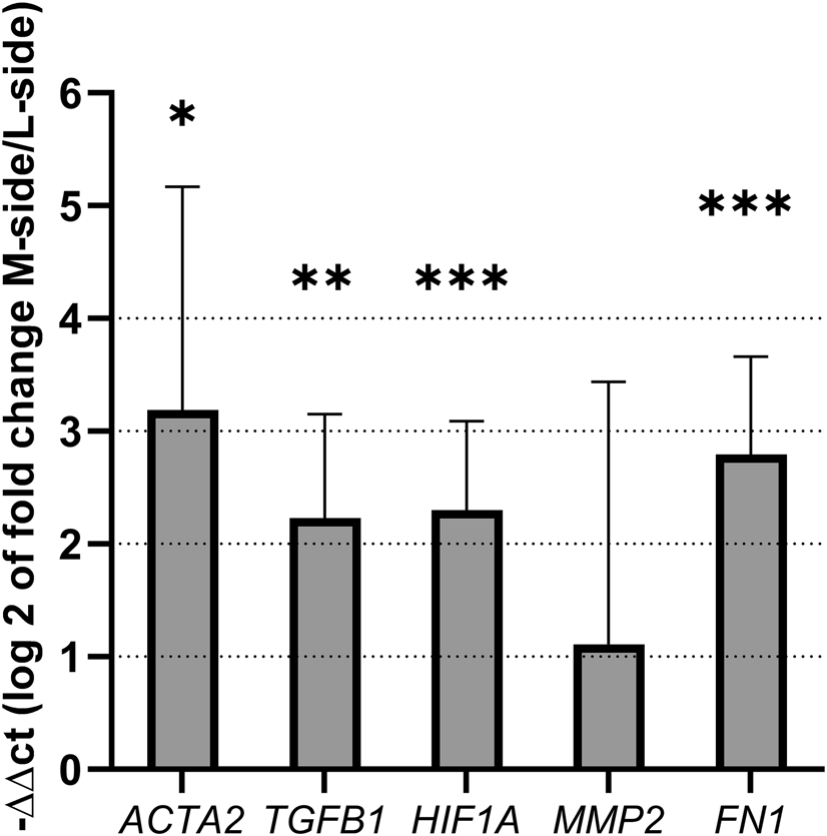
The fold change in the relative mRNA expression of selected genes (*ACTA2*, *TGFB1*, *HIF1A*, *MMP2*, *FN1*) in the M-side versus the L-side of the relapsed clubfoot tissue. A significant increase in expression was detected in the contracted medial side, in comparison with the non-contracted lateral side. The gene expression is normalized to the reference gene *B2M* and the mean expression value of the particular gene in the corresponding L-side tissue sample. Data are presented on a log scale as mean ± SD. The significance level is indicated as follows: *p < 0.05, **p < 0.01, ***p < 0.005.

There was an increase in the relative mRNA expression of protein markers connected with tissue hypoxia in the M-side. A significant increase was found for *ACTA2* (p = 0.0322), *TGFB1* (p = 0.0083), *HIF1A* (p = 0.0043) and *FN1* (p = 0.003). *MMP2* was the only gene, the increase of which was without significance (p = 0.346) (Fig. 3). The gene expression of *MMP9* was also tested; however, the concentration of its mRNA in the tissue samples was relatively low, and also the expression level (especially in the case of L-side samples, where it was below the detection limit in most samples). Although an increase in *MMP9* was found in some M-side samples, we decided not to evaluate (results not shown) and to show where this increase was actually detected. All of these results are in full agreement with the results acquired on the protein level.

### MS label-free quantification

Hemoglobin subunit alpha and hemoglobin subunit beta (HBA1, HBB) were detected as significantly overexpressed in the contracted M-side in comparison with the L-side of the relapsed clubfoot tissue by means of MS label-free quantification (Table 2). In addition, four other proteins (collagen types III and VI, transforming growth factor-beta inducible protein, and tenascin C) were found in significantly higher concentrations in the M-side (all FDR p ≤ 0.05; n = 10) (Table 2). The relative concentration of these proteins in the tissue was not sufficiently high, and therefore our label-free MS quantification allowed us to compare only about 50 of the most abundant proteins. These data were acquired as part of a broad range proteomic analysis of M-side and L-side tissues, revealing mainly fibroproliferative changes in the M-side, similar to the changes that we had found earlier in a different group of samples^7^. The quantitative changes in the hemoglobin subunits are presented here in the context of hypoxic processes. The other hypoxic proteins were below the detection limit of our label-free MS spectrometer and are therefore quantified in this study by IHC.

**Table 2:** Results of label-free MS of hemoglobin subunits alpha and beta with a significant overexpression in the contracted M-side, in comparison with the non-contracted L-side of the relapsed clubfoot tissue.

Accession number	Protein upregulated in M-side	Total number of peptides	Number of significantly different peptides	FDR adjusted p-value	M/L (fold)
P12109	Collagen alpha-1(VI) chain	92	11	0.0008	1.32
P68871	HBB hemoglobin subunit beta	10	5	0.0016	1.62
Q15582	Transforming growth factor-beta-induced protein	4	3	0.0036	3.33
P69905	HBA1 hemoglobin subunit alpha	10	4	0.00324	1.50
P69905	Collagen alpha-1(III) chain	126	11	0.0342	1.12
P24821	Tenascin C	8	0	0.0468	1.28

The total number of peptides indicates the number of successfully compared tryptic peptides detected in at least 50% of all samples. Specific accession numbers of proteins are used from the UNIPROT database (www.uniprot.org) and M/L (fold change) symbolizes the ratio of the protein concentration (M-side/L-side).

### Protein enrichment analysis

The relevant functions of 13 proteins (collagen types I, III, V, and VI, tenascin C, fibronectin, TGF-β, TGF-βIP, HIF1A, MMP-2, MMP-9, hemoglobin alfa and hemoglobin beta) were revealed by the computational prediction of Enrichr. These proteins were selected for this enrichment analysis, because they were several times detected (by MS and/or by IHC and/or by expression of their genes by PCR) as upregulated to the M-side in the present study, and/or in our two recent papers^7,14^. Significant connections of these proteins to hypoxia pathways were found in both Enrichr categories: (i) “Panther 2016” with the result: “Hypoxia response via HIF activation” (FDR adjusted p-value = 0.016) and (ii) “GO:0015671” with the result: “Oxygen transport” (FDR adjusted p-value = 0.00020). The protein–protein interaction (PPI) was also analyzed by the Search Tool for the Retrieval of Interacting Genes and Proteins (STRING) (the same 13 proteins were included), and the PPI enrichment p-value was less than 1.0^−16^ (Supplement 1).

## Discussion

**Figure 4:**
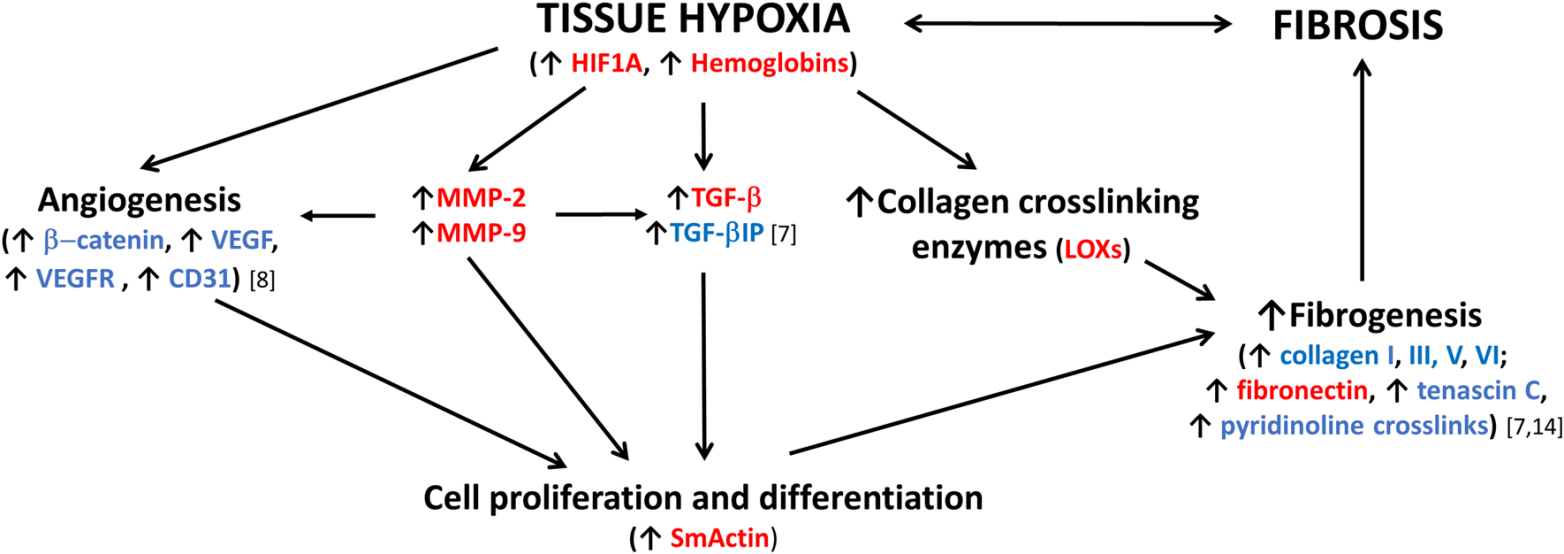
Diagram of protein interactions involved in fibrosis formation in (relapsed) clubfoot. Marked proteins were found significantly upregulated (red—in this study; blue—in this study and/or in our other studies^7,8,14^. *SmActin* alpha smooth muscle actin, *HIF1A* hypoxia-inducible factor 1 alpha, *MMP-2, MMP-9* matrix metalloproteinase-2 and 9, *LOXs* lysyl oxidases, *TGF-β* transforming growth factor-beta, *TGF-βIP* transforming growth factor-beta-induced protein, *VEGF* vascular endothelial growth factor, *VEGFR* vascular endothelial growth factor receptor.

To date, only a small number of studies of the etiology of clubfoot relapses after treatment with the Ponseti method have been reported^15^. Professor Ponseti outlined an increase in collagen synthesis as a common pathological factor in relapses after clubfoot therapy^16^. We therefore decided to pursue this topic in greater depth. An increase in the production of collagens and angioproliferation as a dominant change in the ECM in the contracted tissue of relapsed clubfoot had been already established^7,8^. The data presented in this study expand on previous findings with evidence of increased expression of substances directly linked with the processes mentioned above. We have focused on analyzing the role of tissue hypoxia as a potential factor inducing these changes. The schematic diagram summarizing the data obtained in this study and also in our previous studies (Fig. 4) presents a simplified overview of individual protein interactions. HIF1A stands in the center as a main regulator of an adaptive response to tissue hypoxia^17^. The proposed schematic interplay between hypoxia, angiogenesis and fibrogenesis are based on similar well-described processes in other diseases, mainly with fibroproliferation and angioproliferation, reported in Dupuytren’s contracture^9^, kidney fibrosis^11^, liver fibrosis^12^ and pulmonary hypertension^18,19^.

We have detected an increase in HIF1A nuclear positivity in the contracted tissue of the relapsed clubfoot by means of IHC and also an increase in its mRNA expression, as detected by Real-time PCR (Figs. 2a, 3). HIF1A is considered to be a marker of ongoing hypoxia^17^, and a potent regulator of many genes, including VEGF and MMPs^20^, influencing vascular remodeling and proliferation.

In our previous study^8^, we detected an increase in microvessel and arteriole density in the contracted tissue of the relapsed clubfoot, accompanied by an overexpression of angioproliferation-related proteins, such as platelet-endothelial cell adhesion molecule (PECAM-1, also known as CD31, encoded by the *PECAM1* gene), vascular endothelial growth factor (VEGF, encoded by *VEGF* gene) and vascular endothelial growth factor receptor (VEGFR-2, encoded by the *VEGFR2* gene). A significant increase in *PECAM1* and *VEGF* gene expression (more than double in comparison with control), correlates fully with our results ascertained in the recent study, and supports the existence of tissue hypoxia in the M-side. In tumorous and inflammatory hypoxic circumstances, the *VEGF* is a target gene for HIF1, and the expression of the VEGF is associated with CD31 angiogenesis^21^.

Increased angiogenesis could also be indirectly promoted by MMP enzymes. Although their primary role lies in the degradation of excessive collagen matrix, these enzymes are considered to be effective promotors of cellular proliferation, migration and differentiation^22–24^. The specific procollagen type I fragments cleaved by MMP-2 and MMP-9 serve as proangiogenic signaling molecules and therefore participate in vascularization and vascular tissue remodeling^24^. The influence of hypoxia on MMPs would probably be specific for each tissue and cell in a specific organism. It was proven that hypoxia activates MMP2 expression in vascular and retinal endothelial cells^25^. Four-day hypoxia also significantly increased the MMP-9 concentration in smooth muscle cells in pulmonary arteries^26^. The role of MMPs in pulmonary hypoxia has been reviewed in^27^. We have detected an increase in MMP-2 and MMP-9 IHC positivity in the contracted M-side tissue of relapsed clubfoot, though without a significant increase in the expression of *MMP2* or *MMP9* genes (Figs. 2b, 3).

MMPs together with HIF1A stimulate the production of TGF-β^28,29^—a cytokine which can modulate fibroblast phenotype and which functions via induction of myofibroblast transdifferentiation^30^. We have detected an increase in TGF-β IHC positivity and also an increase in the mRNA of *TGFB1* gene expression (Figs. 2a, 3). A possible increase in myofibroblast number was suggested by the detected increase in the positivity of SmActin in the contracted tissue of relapsed clubfoot by means of IHC, and also by an increase in *ACTA2* gene expression (Figs. 2a, 3). SmActin is considered to be a marker of mature myofibroblasts^31^. Active myofibroblasts excessively produce collagens and other components of ECM, and cause contraction of the tissue^32^. These data are consistent with the hypothesis of hypoxic fibrosis in relapsed clubfoot (Fig. 4).

In our previous study, we detected changes in the composition of the ECM in the contracted M-side tissue of relapsed clubfoot—an increase in collagen III, V and VI, TGF-βIP, asporin, tenascin C, and qualitative changes in the distribution of TGF-β^7^. Additionally, we confirmed significant changes in the levels of four of these proteins (collagen types III and VI, TGF-βIP, and tenascin C) on a new set of samples in the study presented here (n = 10) (Table 2), together with an increase in the positivity of fibronectin by means of IHC and an increase in *FN1* gene expression (Figs. 2b, 3). The altered composition of the ECM and the altered levels of ECM proteins point to the existence of fibrotic changes in the contracted tissue of relapsed clubfoot.

Fibronectin gene transcription is stimulated by beta-catenin signaling^33^. The active form of beta-catenin, which was also found upregulated in clubfoot M-side^8^, is an important modulating element in angiogenesis that enhances endothelial cell proliferation and the induction of VEGF expression^34^, which could point to a link in the interaction between hypoxia-induced angiogenesis and fibrogenesis (Fig. 4).

Tenascin C is a matrix protein that regulates the interactions of cells with matrix components and growth factors. It has an important role during fibrogenesis, as it activates the TGF-ß signaling pathway, through which it influences the myofibroblast differentiation as well as the activation of matrix metalloproteinases (reviewed in^35^). The upregulation of tenascin C in the medial part of clubfoot adds further evidence of fibrotic changes in this tissue.

Tissue hypoxia is also connected with the upregulation of LOXs activity. LOXs are amine oxidases required for biosynthetic cross-linking of ECM components, and they therefore affect final fibrogenesis^36^. It has been observed that the expression of *LOX* and *LOXL2* genes is positively regulated by HIF^36,37^. This regulatory mechanism has been associated with hypoxia and tumor progression, and has been shown to stimulate tumor cell motility and migration in breast and colorectal cancer^38,39^ (reviewed in^40^). We observed a significant increase in the positivity of LOX and LOXL2 (two of the main LOXs) in the contracted tissue (M-side) of the relapsed clubfoot in comparison with non-contracted tissue by IHC (Fig. 2a). These results are in good agreement with our recent study, where we demonstrated significant upregulation of the most common collagen crosslinks (pyridinoline and deoxypyridinoline, which are terminal products of LOXs) in the clubfoot M-side (as compared with the L-side) tissue^14^. Accumulation of these crosslinks impairs normal collagen degradability and contributes to greater tissue stiffness. These data are consistent with the pro-fibrotic theory of clubfoot relapse, and are also in agreement with the presence of hypoxia during this disease (Fig. 4).

The elevation of hemoglobin subunits alpha and beta detected by Mass Spectrometry in the contracted tissue of relapsed clubfoot may also be a part of a pathway related to tissue hypoxia^41^. This may also be connected with ECM remodeling, as suggested by previously detected changes in ECM protein content^7^. It has been shown previously that hemoglobin subunits alpha and beta are also produced in endothelial cells^42,43^. Hemoglobin subunit alpha plays a role in the regulation of nitric oxide signaling via myoendothelial junction. This pathway may also play a role in regulating the blood flow in arteries^42^. In the field of cancer research, an overexpression of hemoglobin subunit beta has been associated with an increase in the level of neoangiogenesis^44^. We therefore conclude that this elevated expression can be caused by an increase in the number of endothelial cells in the new vessels.

Moreover, the computational prediction of Enrichr performed with 13 proteins (collagen types I, III, V, and VI, tenascin C, fibronectin, TGF-β, TGF-βIP, HIF1A, MMP-2, MMP-9, along with hemoglobin alfa and hemoglobin beta) revealed their significant connection to hypoxia pathways. Our results are in agreement with the data of a recent integrated bioinformatic analysis of clubfoot disease by Cai et al.^45^, who found that some of the signaling pathways in clubfoot are involved in “the regulation of gene expression by a hypoxia-inducible factor” and in the “cell response to hypoxia” (these pathways were enriched in the REAC database).

We are aware that our study has specific limitations that do not allow us to prove conclusively that its results are applicable to the non-relapsed clubfoot in its entirety. All samples used in our study were taken from patients who had undergone surgical treatment due to a relapse after unsuccessful casting therapy. Although we fully understand the risk involved in the potential role of repeated castings and of consequent bruising of the tissues of the treated clubfoot, there is unfortunately no way for us to obtain tissue samples from previously untreated patients. Similarly, obtaining tissue samples from treated but non-relapsed patients of similar age to be used as a control group presents a serious ethical dilemma and it is near impossible to obtain the necessary parental consent for children’s participation in the research. The same applies for gaining access to tissue from healthy pediatric donors (i.e. a group of patients without clubfoot). Therefore, the most feasible option for examining relevant pathological tissue from clubfoot under these circumstances is to compare differences between the tissue from the contracted M-side and from the non-contracted L-side of the relapsed clubfoot—at least until a suitable biological model of clubfoot emerges, as has happened e.g. with Dupuytren’s contracture^46^.

We believe that the insights into the hypoxia-induced pathway, suggested by our findings, provide valuable information for future studies, which could subsequently provide conclusive evidence. We also believe that there may be a translational potential, which could lead in the future to the detection of therapeutic targets at the level of disease onset and progression. This could shift clubfoot therapy from symptomatic towards cell-targeted, as has already been described in other fibroproliferative disorders^47^. In addition, the reported stiffness of the fibrotic clubfoot tissue might be alleviated in the future by the application of antifibrotic substances, for example targeting ECM crosslinking enzymes^14,48^. Such antifibrotic treatments would facilitate the currently-used conservative therapy by making the collagens of the affected tissue more susceptible to degradation, and thus by making the tissue less stiff.

## Conclusion

In our study we have detected hypoxia-related proteins in the contracted medial side of relapsed clubfoot, and we have presented a possible connection between these newly-detected proteins and previously-detected fibrosis and angiogenesis in this tissue. Hypoxic pathways are directly related to both fibrosis and angiogenesis, i.e. processes which also alter the ECM in the affected tissue of relapsed clubfoot (Fig. 4). Our findings are a contribution to knowledge of the etiology and pathogenesis of relapses in clubfoot therapy, and potentially a contribution to the development of a strategy for clubfoot therapy in the future.

## Materials and methods

### Biological material

Ten patients (nine boys, one girl; mean age of 53.4 months) with idiopathic clubfoot were initially treated with the Ponseti method. In these ten children with relapsed clubfoot, tissue samples were obtained at the time of surgery for relapsed clubfoot, using previously published methods^7,8^.

All procedures were performed in accordance with the ethical standards in the 1964 Declaration of Helsinki. Institutional approval for the present study was obtained from the Ethics Committee of the Institute of Physiology of the Czech Academy of Sciences (project No. 17-31564A, 14th June 2016). The parents or legal guardians of all patients provided written, informed consent to participate. This study is analytical, prospective, level of evidence IIb.

### Histopathological investigation

Tissue samples, both medial and lateral, of all 10 patients were fixed and embedded in paraffin. Primary antibodies against alpha smooth muscle actin (anti-SmActin, A5228, Sigma; 1:400, 4 °C overnight), transforming growth factor-beta (anti-TGF-β, ab66043, Abcam; 1:200, 4 °C overnight), hypoxia-inducible factor 1 alpha (anti-HIF1A, ab216842, Abcam; 1:200, 1 h RT), ), lysyl oxidase (anti-LOX, NB 100-2527, Novus Biologicals, 1:200, 2 h RT), lysyl oxidase-like 2 (anti-LOXL2, NBP1-32,954, Novus Biologicals, 1:100, 4 °C overnight), tenascin C (anti-TN-C, ab3970, Abcam, 1:200, 1 h RT), matrix metalloproteinase-2 (anti-MMP-2, ab37150, Abcam; 1:500, 15 min RT), matrix metalloproteinase-9 (anti-MMP-9 (ab73734, 1:181, 15 min RT), fibronectin (anti-Fibronectin, ab2413, Abcam; 1:100, 1 h RT) were used for detection by immunohistochemistry (IHC). Antigen retrieval, hydrogen peroxide block, protein block, secondary antibody reaction, and visualization were performed according to the Abcam protocol by applying the EXPOSE Mouse and Rabbit Specific HRP/DAB IHC Detection Kit (ab236466, Abcam, Cambridge, UK). The slides were counterstained with hematoxylin. The positivity of the detected antibodies was evaluated by a light microscope and the quantification was performed using image analyzer signal thresholding (NIS Elements 3.0 AR, Laboratory Imaging, Czech Republic). We used a positive control (a tissue selected according to the recommendation of the antibody producer) for manually defined thresholding of the positive signal. The number of pixels within the signal range was then quantified from 10 independent areas of each sample, and the percentage of the positive area was calculated and was compared across the experimental groups. For each experimental group, we followed the recommendation of Johnson et al*.* that the same primary and secondary antibodies should be applied to all tissues, the same reagents should be used at the same concentrations, and that all incubation and development times should be identical^49^. For the purposes of the image analysis, no deconvolution was used. Both object classes (negative vs. positive) were segmented using a threshold in RGB colour space.

### Total mRNA isolation

The samples which were used for mRNA analyses were cut from the original tissue at the time of surgery, were frozen at − 80 °C, and were processed separately from the IHC samples. The total mRNA was isolated using the Animal Tissue RNA Purification Kit (Norgen Biotek). Tissue samples for total mRNA isolation (n = 10 for M-side, n = 10 for L-side) 20 mg in weight were placed into 2.0 ml sample tubes filled with 0.5 ml of cold lysis buffer and ceramic beads for homogenizing the solid cellular sample material (MagNA Lyser Green Beads – Roche), and were homogenized three times for 30 s (speed 10,000 rpm) by a Precellys Evolution homogenizer (Bertin Instruments, Montigny-le-Bretonneux, France). Isolation was then carried out according to the manufacturer’s protocol. These methods are described in our previous work^8^.

### Real-time PCR

Real-time PCR was performed to investigate the relative mRNA expression of protein markers connected with hypoxia. Reverse transcription was performed using the Omniscript Reverse Transcription Kit (205113; Qiagen, Hilden, Germany) and random hexamers (New England Biolabs, Inc, Ipswich, MA, USA), and was carried out according to the manufacturer’s protocol. The mRNA level was quantified using 5 × HOT FIREPol Probe qPCR Mix Plus (ROX) (08-36-00001; Solis BioDyne, Tartu, Estonia) and by TaqMan Gene Expression Assays (4331182; Thermo Fisher Scientific), labeled with FAM reporter dye specific to human genes: *ACTA2* (smooth muscle α-actin, Hs00909499_m1), *FN1* (fibronectin 1, Hs01549976_m1), *TGFB1* (transforming growth factor-beta 1, Hs0177257_m1), *HIF1A* (hypoxia-inducible factor, Hs00153153_m1) and *MMP2* (matrix metalloproteinase-2, Hs001548724_m1). The experiments were performed with *B2M* as a reference gene (B2 microglobulin, Hs00187842_m1) using the Viia 7 Real-time PCR System (Applied Biosystems; Thermo Fisher Scientific) in a 96-well optical reaction plate.

### Mass spectrometry quantification

Samples of approximately 10 mg dry weight (n = 10 for M-side, n = 10 for L-side) were processed for mass spectrometry (MS), as described in our previous study^7^. The Nano-liquid chromatography procedure, MS and tandem MS (MS/MS) analyses were performed as described in our previous studies^7,8^, with upgraded software. Database searches were carried out using the Uniprot databases (uniprot.org) with the taxonomy restricted to Homo sapiens. When analyzing MS label-free quantification, only significant hits (MASCOT score ≥ 80 for proteins; ≥ 30 for peptides, http://www.matrixscience.com) were accepted.

### Protein enrichment analysis

The computational prediction of Enrichr (https://maayanlab.cloud/Enrichr/enrich#) was used to provide a functional interpretation of the predicted potential targets of the protein in the specific enrichment categories according to “Gene Ontology (GO) Biological Process” and “Panther”. A specific organism was chosen: *H. sapiens*. Relevant functions of a total of 13 proteins were identified. These proteins were detected as upregulated in the contracted M-side of clubfoot (proteins from the present study and proteins from our two recent papers)^7,14^. The protein–protein interaction (PPI) was analyzed by the Search Tool for the Retrieval of Interacting Genes and Proteins (STRING; http://string-db.org) for the same group of proteins.

### Statistical analysis

#### IHC

The IHC positive area data from both sides were examined for normality (Q-Q plot) and were compared using the Student t-test for two sampled (non-paired) data. The significance level for rejection of the null hypothesis was set at 0.05.

#### Real-time PCR

The relative gene expression was calculated as 2^−ΔΔCt^. The data are reported as the ratio M-side/L-side presented as mean ± SD. The data were normalized against the average of the L-side group for each patient. The results are the means of 20–24 experimental points acquired from 10 sampled tissue pieces. The data were tested for normality by the Shapiro–Wilk test. Statistical significance was analyzed by a one-sample t-test. The significance level for rejection of the null hypothesis was set at 0.05.

#### Mass spectrometry quantification

Profile Analysis software (Bruker) was used to evaluate the differences in the protein composition of the L-side and the M-side, by means of MS label-free quantification. The peptides under consideration had to be found in at least 50% of all samples, regardless of the group, and at the same time they had to be found in at least one of the two groups (L or M), and also in at least 50% of the samples of the group. The p-values given by two-sample t-tests were corrected for multiple-testing by the false discovery rate (FDR) based on a frequency histogram (FDR adjusted p-value threshold 0.05).

## Supplementary Information

Supplementary Information.

## Data Availability

The datasets generated during and/or analysed during the current study are available from the corresponding author on reasonable request.
