# Supplementary material for: The possible role of hypoxia in the affected tissue of relapsed clubfoot
Source: Sci Rep. 2022 Mar 15;12:4462. doi: 10.1038/s41598-022-08519-z (PMC8924187; doi:10.1038/s41598-022-08519-z)
Supplement: Supplementary file 1 — Supplementary Information. [file 41598_2022_8519_MOESM1_ESM.pdf]

Version: **11.5**

LOGIN REGISTER

Search Download Help My Data

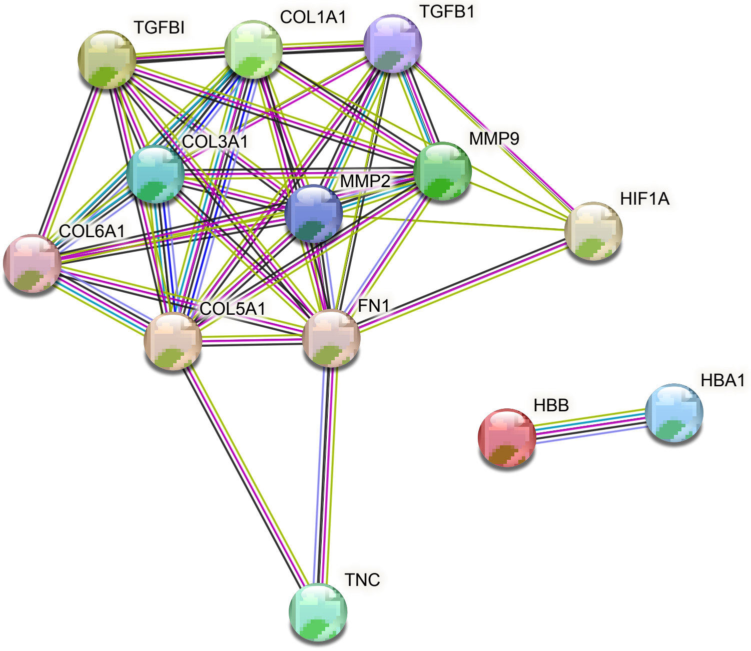

Viewers Legend Settings Analysis Exports Clusters More Less

Network Stats

number of nodes:	13	expected number of edges:	5
number of edges:	43	PPI enrichment p-value:	< 1.0e-16
average node degree:	6.62	*your network has significantly more interactions than expected (what does that mean?)*	
avg. local clustering coefficient:	0.915		

Functional enrichments in your network

explain columns

Biological Process (Gene Ontology)

GO-term	description	count in network	strength	false discovery rate
GO:0002248	Connective tissue replacement involved in inflammatory res…	2 of 5	2.78	0.0032
GO:0001957	Intramembranous ossification	2 of 6	2.7	0.0038
GO:0060346	Bone trabecula formation	2 of 8	2.58	0.0052
GO:0032964	Collagen biosynthetic process	2 of 9	2.52	0.0057
GO:0035987	Endodermal cell differentiation	4 of 41	2.17	2.48e-05
GO:0071295	Cellular response to vitamin	2 of 24	2.1	0.0249
GO:0032963	Collagen metabolic process	5 of 63	2.08	1.07e-06
GO:0070168	Negative regulation of biomineral tissue development	2 of 28	2.03	0.0313
GO:0042744	Hydrogen peroxide catabolic process	2 of 28	2.03	0.0313
GO:0010575	Positive regulation of vascular endothelial growth factor pro…	2 of 28	2.03	0.0313
GO:0030199	Collagen fibril organization	3 of 46	1.99	0.0016
GO:0060325	Face morphogenesis	2 of 31	1.99	0.0354
GO:0018149	Peptide cross-linking	2 of 34	1.95	0.0410
GO:0071230	Cellular response to amino acid stimulus	4 of 69	1.94	9.86e-05
GO:1904707	Positive regulation of vascular associated smooth muscle c…	2 of 35	1.93	0.0430
GO:0071634	Regulation of transforming growth factor beta production	2 of 39	1.89	0.0484
GO:1902895	Positive regulation of pri-mirna transcription by rna polymer…	2 of 40	1.88	0.0494
GO:0007566	Embryo implantation	2 of 41	1.87	0.0496
GO:0002062	Chondrocyte differentiation	3 of 84	1.73	0.0060
GO:0048771	Tissue remodeling	3 of 96	1.67	0.0081
GO:0030198	Extracellular matrix organization	10 of 338	1.65	1.32e-11
GO:0051216	Cartilage development	5 of 171	1.64	6.55e-05
GO:0061448	Connective tissue development	6 of 223	1.61	6.12e-06
GO:0000302	Response to reactive oxygen species	5 of 198	1.58	9.86e-05
GO:0042542	Response to hydrogen peroxide	3 of 119	1.58	0.0139
GO:0001649	Osteoblast differentiation	3 of 127	1.55	0.0157
GO:0030168	Platelet activation	3 of 135	1.52	0.0183
GO:0010634	Positive regulation of epithelial cell migration	3 of 147	1.49	0.0232
GO:0071560	Cellular response to transforming growth factor beta stimulus	3 of 149	1.48	0.0238
GO:0048762	Mesenchymal cell differentiation	3 of 152	1.47	0.0249
GO:0001503	Ossification	5 of 265	1.45	0.00033
GO:0042060	Wound healing	8 of 439	1.44	4.71e-07

GO:0042060	Wound healing	[illegible]	[illegible]	[illegible]
GO:0001568	Blood vessel development	8 of 500	1.38	9.27e-07
GO:0001501	Skeletal system development	8 of 499	1.38	9.27e-07
GO:2000377	Regulation of reactive oxygen species metabolic process	3 of 188	1.38	0.0398
GO:0006979	Response to oxidative stress	6 of 393	1.36	8.75e-05
GO:0048514	Blood vessel morphogenesis	6 of 410	1.34	9.86e-05
GO:0007162	Negative regulation of cell adhesion	4 of 280	1.33	0.0068
GO:0009612	Response to mechanical stimulus	3 of 212	1.33	0.0494
GO:0001525	Angiogenesis	4 of 315	1.28	0.0100
GO:0035239	Tube morphogenesis	7 of 656	1.21	6.55e-05
GO:0048729	Tissue morphogenesis	6 of 561	1.21	0.00044
GO:0048598	Embryonic morphogenesis	6 of 571	1.2	0.00046
GO:0030335	Positive regulation of cell migration	5 of 522	1.16	0.0046
GO:0007507	Heart development	5 of 522	1.16	0.0046
GO:0010035	Response to inorganic substance	5 of 538	1.15	0.0050
GO:0002009	Morphogenesis of an epithelium	4 of 435	1.14	0.0277
GO:0048646	Anatomical structure formation involved in morphogenesis	8 of 883	1.13	2.48e-05
GO:0009887	Animal organ morphogenesis	8 of 967	1.1	4.42e-05
GO:0050878	Regulation of body fluid levels	4 of 509	1.07	0.0435
GO:0007169	Transmembrane receptor protein tyrosine kinase signaling …	4 of 518	1.07	0.0453
GO:0007155	Cell adhesion	7 of 925	1.06	0.00041
GO:0070848	Response to growth factor	4 of 524	1.06	0.0457
GO:0019221	Cytokine-mediated signaling pathway	5 of 678	1.05	0.0116
GO:0009790	Embryo development	7 of 1002	1.02	0.00058
GO:0030334	Regulation of cell migration	6 of 865	1.02	0.0035
GO:0030155	Regulation of cell adhesion	5 of 712	1.02	0.0140
GO:0007167	Enzyme linked receptor protein signaling pathway	5 of 720	1.02	0.0146
GO:1901701	Cellular response to oxygen-containing compound	7 of 1055	1.0	0.00078
GO:0008284	Positive regulation of cell population proliferation	6 of 919	0.99	0.0045
GO:0034097	Response to cytokine	7 of 1101	0.98	0.0010
GO:0009888	Tissue development	11 of 1760	0.97	9.27e-07
GO:0071345	Cellular response to cytokine stimulus	6 of 1013	0.95	0.0060
GO:1901700	Response to oxygen-containing compound	9 of 1567	0.94	6.55e-05
GO:0016477	Cell migration	5 of 896	0.92	0.0328
GO:0009653	Anatomical structure morphogenesis	11 of 2165	0.88	3.45e-06
GO:0071495	Cellular response to endogenous stimulus	6 of 1181	0.88	0.0127
GO:0051093	Negative regulation of developmental process	5 of 983	0.88	0.0453
GO:0010243	Response to organonitrogen compound	5 of 987	0.88	0.0454
GO:0051241	Negative regulation of multicellular organismal process	6 of 1231	0.87	0.0151
GO:0071310	Cellular response to organic substance	9 of 2369	0.76	0.0011
GO:0070887	Cellular response to chemical stimulus	11 of 2919	0.75	4.97e-05
GO:0045595	Regulation of cell differentiation	7 of 1874	0.75	0.0155
GO:0048513	Animal organ development	11 of 3197	0.71	9.86e-05
GO:2000026	Regulation of multicellular organismal development	7 of 2096	0.7	0.0281
GO:0006950	Response to stress	11 of 3485	0.68	0.00020
GO:0007166	Cell surface receptor signaling pathway	7 of 2325	0.66	0.0454
GO:0030154	Cell differentiation	10 of 3702	0.61	0.0035
GO:0032501	Multicellular organismal process	12 of 6933	0.42	0.0081
GO:0050896	Response to stimulus	13 of 8046	0.39	0.0035
GO:0008152	Metabolic process	12 of 8298	0.34	0.0430

(less ...)

Molecular Function (Gene Ontology)

GO-term	*description*	*count in network*	*strength*	*false discovery rate*
GO:0048407	Platelet-derived growth factor binding	4 of 11	2.74	4.15e-07
GO:0030020	Extracellular matrix structural constituent conferring tensile …	4 of 28	2.33	7.24e-06
GO:0005344	Oxygen carrier activity	2 of 14	2.33	0.0282
GO:0043394	Proteoglycan binding	3 of 37	2.09	0.0017
GO:0005201	Extracellular matrix structural constituent	7 of 119	1.95	3.35e-09

(more ...)

Cellular Component (Gene Ontology)

GO-term	*description*	*count in network*	*strength*	*false discovery rate*
GO:0005583	Fibrillar collagen trimer	3 of 12	2.58	4.52e-05
GO:0031838	Haptoglobin-hemoglobin complex	2 of 11	2.44	0.0065
GO:0005833	Hemoglobin complex	2 of 12	2.4	0.0070
GO:0071682	Endocytic vesicle lumen	2 of 20	2.18	0.0127
GO:0005581	Collagen trimer	4 of 88	1.84	9.70e-05

(more ...)

Reference publications (PubMed)

publication	*(year) title*	*count in network*	*strength*	*false discovery rate*
PMID:31086584	(2019) Investigation of expression and effects of TGF-Beta…	4 of 4	3.18	3.44e-07
PMID:30321755	(2019) Effect of transforming growth factor -beta1 on alpha-…	4 of 4	3.18	3.44e-07
PMID:16640888	(2006) [Interactions of ovarian carcinoma cells and human …	4 of 4	3.18	3.44e-07
PMID:10712429	(2000) Hypoxia-inducible factor-1 mediates the biological ef…	4 of 4	3.18	3.44e-07
PMID:9950685	(1999) Novel regulation of type IV collagenase (matrix meta…	3 of 3	3.18	2.93e-05

(more ...)

Local network cluster (STRING)

cluster	*description*	*count in network*	*strength*	*false discovery rate*
CL:16430	Collagen formation, and Matrix metalloproteinases	6 of 129	1.85	0.00079

	KEGG Pathways			
pathway	description	count in network	strength	false discovery rate
hsa04933	AGE-RAGE signaling pathway in diabetic complications	5 of 98	1.89	1.03e-06
hsa05219	Bladder cancer	2 of 41	1.87	0.0149
hsa04512	ECM-receptor interaction	4 of 88	1.84	3.59e-05
hsa05144	Malaria	2 of 46	1.82	0.0171
hsa05146	Amoebiasis	4 of 100	1.78	4.70e-05

(more ...)

	Reactome Pathways			
pathway	description	count in network	strength	false discovery rate
HSA-1247673	Erythrocytes take up oxygen and release carbon dioxide	2 of 8	2.58	0.0132
HSA-3000170	Syndecan interactions	6 of 27	2.52	4.19e-10
HSA-1237044	Erythrocytes take up carbon dioxide and release oxygen	2 of 12	2.4	0.0235
HSA-2168880	Scavenging of heme from plasma	2 of 13	2.36	0.0246
HSA-8874081	MET activates PTK2 signaling	4 of 30	2.3	9.31e-06

(more ...)

	WikiPathways			
pathway	description	count in network	strength	false discovery rate
WP3967	miR-509-3p alteration of YAP1/ECM axis	4 of 17	2.55	1.21e-06
WP2911	miRNA targets in ECM and membrane receptors	4 of 22	2.44	1.51e-06
WP3859	TGF-beta signaling in thyroid cells for epithelial-mesenchy…	3 of 18	2.4	0.00011
WP3670	Interactions between LOXL4 and oxidative stress pathway	2 of 18	2.22	0.0103
WP453	Inflammatory response pathway	3 of 30	2.18	0.00027

(more ...)

	Disease-gene associations (DISEASES)			
disease	description	count in network	strength	false discovery rate
DOID:0110031	Hemoglobin h disease	2 of 5	2.78	0.0034
DOID:12169	Carpal tunnel syndrome	2 of 8	2.58	0.0057
DOID:90	Degenerative disc disease	3 of 13	2.54	0.00028
DOID:13359	Ehlers-Danlos syndrome	3 of 23	2.29	0.00038
DOID:10126	Keratoconus	2 of 18	2.22	0.0177

(more ...)

	Tissue expression (TISSUES)			
tissue	description	count in network	strength	false discovery rate
BTO:0000140	Bone	5 of 138	1.74	1.80e-05
BTO:0001078	Placenta	9 of 1176	1.06	1.80e-05
BTO:0001486	Skeletal system	9 of 1203	1.05	1.80e-05
BTO:0000421	Connective tissue	6 of 871	1.02	0.0028
BTO:0000439	Eye	5 of 786	0.98	0.0207

(more ...)

	Subcellular localization (COMPARTMENTS)			
compartment	description	count in network	strength	false discovery rate
GOCC:0098643	Banded collagen fibril	3 of 11	2.61	3.86e-05
GOCC:0005583	Fibrillar collagen trimer	3 of 11	2.61	3.86e-05
GOCC:0031838	Haptoglobin-hemoglobin complex	2 of 11	2.44	0.0074
GOCC:0098644	Complex of collagen trimers	3 of 20	2.35	0.00014
GOCC:0005581	Collagen trimer	4 of 35	2.24	5.30e-06

(more ...)

	Annotated Keywords (UniProt)			
keyword	description	count in network	strength	false discovery rate
KW-0248	Ehlers-Danlos syndrome	3 of 17	2.42	5.56e-05
KW-0177	Collagen degradation	2 of 18	2.22	0.0071
KW-0176	Collagen	4 of 82	1.87	5.56e-05
KW-0272	Extracellular matrix	10 of 265	1.75	6.46e-14
KW-0379	Hydroxylation	5 of 140	1.73	7.86e-06

(more ...)

	Protein Domains (Pfam)			
domain	description	count in network	strength	false discovery rate
PF01410	Fibrillar collagen C-terminal domain	3 of 11	2.61	0.0012
PF00040	Fibronectin type II domain	3 of 14	2.51	0.0012
PF01391	Collagen triple helix repeat (20 copies)	4 of 76	1.9	0.0012

	Protein Domains and Features (InterPro)			
domain	description	count in network	strength	false discovery rate
IPR000885	Fibrillar collagen, C-terminal	3 of 11	2.61	0.0024
IPR036943	Fibronectin type II domain superfamily	3 of 13	2.54	0.0024
IPR000562	Fibronectin type II domain	3 of 13	2.54	0.0024
IPR013806	Kringle-like fold	3 of 26	2.24	0.0048
IPR008160	Collagen triple helix repeat	4 of 77	1.89	0.0024

	Protein Domains (SMART)			
domain	*description*	*count in network*	*strength*	*false discovery rate*
SM00059	Fibronectin type 2 domain	3 of 14	2.51	0.00020
SM00038	Fibrillar collagens C-terminal domain	2 of 10	2.48	0.0169
SM00120	Hemopexin-like repeats.	2 of 21	2.16	0.0431

Statistical background

For the above enrichment analysis, the following statistical background is assumed: Whole Genome ADD BACKGROUND UPDATE

Save / Export

Biological Process (Gene Ontology)	download	*81 GO-terms significantly enriched; file-format: tab-delimited*
Molecular Function (Gene Ontology)	download	*9 GO-terms significantly enriched; file-format: tab-delimited*
Cellular Component (Gene Ontology)	download	*14 GO-terms significantly enriched; file-format: tab-delimited*
Reference publications (PubMed)	download	*10000 publications significantly enriched; file-format: tab-delimited*
Local network cluster (STRING)	download	*one single cluster is enriched; file-format: tab-delimited*
KEGG Pathways	download	*13 pathways significantly enriched; file-format: tab-delimited*
Reactome Pathways	download	*23 pathways significantly enriched; file-format: tab-delimited*
WikiPathways	download	*25 pathways significantly enriched; file-format: tab-delimited*
Disease-gene associations (DISEASES)	download	*13 diseases significantly enriched; file-format: tab-delimited*
Tissue expression (TISSUES)	download	*9 tissues significantly enriched; file-format: tab-delimited*
Subcellular localization (COMPARTMENTS)	download	*21 compartments significantly enriched; file-format: tab-delimited*
Annotated Keywords (UniProt)	download	*15 keywords significantly enriched; file-format: tab-delimited*
Protein Domains (Pfam)	download	*3 domains significantly enriched; file-format: tab-delimited*
Protein Domains and Features (InterPro)	download	*5 domains significantly enriched; file-format: tab-delimited*
Protein Domains (SMART)	download	*3 domains significantly enriched; file-format: tab-delimited*

Server load: low (7%) [HD]

Permalink

© STRING CONSORTIUM 2021

SIB - Swiss Institute of Bioinformatics

CPR - Novo Nordisk Foundation Center Protein Research

EMBL - European Molecular Biology Laboratory

ABOUT
Content
References
Contributors
Statistics

INFO
Scores
Use scenarios
FAQs
Cookies/Privacy

ACCESS
Versions
APIs
Licensing
Usage

CREDITS
Funding
Datasources
Partners
Software

STRING is part of the ELIXIR infrastructure: it is one of ELIXIR's Core Data Resources. Learn more >
